# Allergies/asthma and root resorption: a systematic review

**DOI:** 10.1186/s40510-021-00351-x

**Published:** 2021-03-15

**Authors:** Cibelle Cristina Oliveira dos Santos, Silvio Augusto Bellini-Pereira, Melany Clarissa Gamez Medina, David Normando

**Affiliations:** 1 Department of Orthodontics, Dental School, Federal University of Pará, Belém, Pará Brazil; 2 Department of Orthodontics, Bauru Dental School, University of São Paulo, São Paulo, Brazil

**Keywords:** Allergy, Asthma, Root resorption, Orthodontics

## Abstract

**Background:**

This review synthesizes the available evidence about the predisposition of individuals with asthma or allergies to orthodontically induced inflammatory root resorption (OIIRR) and possible factors related to root resorption that were investigated in the included studies, such as the type of malocclusion, duration of orthodontic treatment, and tooth units.

**Material and methods:**

Six electronic databases and partial gray literature were searched without date or language restrictions until September 2020. Prospective and retrospective observational cohort and case-control studies were included. The risk of bias (RoB) was assessed using the checklists from the Joanna Briggs Institute and the certainty of the evidence using the GRADE tool. To complement the case-control studies, the odds ratio (OR) of the individuals with allergies/asthma to develop root resorption was calculated.

**Results:**

Six studies were included. One study with low RoB, one with moderate, and one with high RoB stated that allergic patients did not report a greater chance of developing OIIRR (OR = 1.17 to 2.10, *p* = 0.1 to 1), while only one study with low RoB reported that individuals with allergies tend to develop root resorption (OR = 2.4, 95% CI = 1.08-5.37). Three studies with low RoB and one with moderate showed no significant association between asthma and OIIRR (OR = 1.05 to 3.42, *p* = 0.12 to 0.94). No association was identified between the type of malocclusion and the degree of OIIRR. Uniradicular dental units and a prolonged treatment time seem to be associated with an increased risk of resorption. The certainty of the evidence was considered low for both exposure factors.

**Conclusion:**

Evidence with a low level of certainty indicates that individuals with allergies or asthma are not more predisposed to OIIRR. Uniradicular teeth and long-term orthodontic treatments are associated with a higher risk of OIIRR.

**Systematic review registration:**

PROSPERO CRD42020188463

## Introduction

Orthodontically induced inflammatory root resorption (OIIRR) affects the apical third and promotes a reduction of approximately 1 mm from the root [1]. It is considered an undesirable and inevitable side effect in approximately 80% of orthodontic patients [2]. However, severe resorption can cause mobility and tooth loss [3]. External factors related to orthodontic mechanics such as the type of appliance [4], intensity and direction of the applied force [4], duration of orthodontic treatment [5], and dental extractions [5] can be associated with OIIRR. Additionally, individual factors such as genetics [6], sex [7], age [8], root morphology [9], bone density [10], and systemic factors related to the immune system [11] were also described as potential factors for OIIRR.

The inflammatory mechanism promoted by immune cells that precede tooth movement can influence the magnitude of root resorption. In patients with asthma, the action of T-helper lymphocytes synthesizes inflammatory mediators that reach the blood circulation and the periodontal ligament interacting with bone remodeling cells and tooth movement [12]. The presence of primary leukocytes in the bloodstream caused by lung diseases supports a possible association between excessive root resorption and pathological conditions that affect the immune system [11]. There is a hypothesis that individuals with allergies or asthma may have a greater chance of developing root resorption after orthodontic treatment [13]. Many of the inflammatory mediators stimulated in an allergic condition, such as asthma, circulate via blood vessels and possibly penetrate the extravascular space of the periodontal ligament, especially during orthodontic tooth movement [14]. A cohort study reported the highest incidence of root resorption in individuals with asthma and concluded that asthma is a risk factor for OIIRR [14]. However, the literature has pointed out divergent results [11, 15]. A retrospective case-control study observed that the prevalence of the allergy risk factor was higher in the group of individuals with root resorption [11]. In contrast, some studies found no association between the presence of the allergy risk factor and a higher level of OIIRR [16, 17].

Orthodontic patients with allergies or asthma are identified before treatment if they have a greater predisposition to the development of root resorption. Considering the inconsistency in the literature on the association between immune diseases and orthodontically induced root resorption, the primary objective of this review was to synthesize the available evidence about the predisposition of individuals with asthma or allergies to orthodontically induced root resorption. The secondary aim was to investigate possible factors related to root resorption that were investigated in the included studies, such as the type of malocclusion, duration of orthodontic treatment, and tooth units.

## Material and methods

### Protocol and registration

This systematic review was submitted to the PROSPERO database (https://www.crd.york.ac.uk/prospero/), under protocol ID CRD42020188463 and carried out according to the PRISMA guidelines (https://Prisma-statement.org/).

### Eligibility criteria

The eligibility criteria were adopted according to the PECOS strategy:

• P: Individuals treated orthodontically.

• E: Allergies or asthma.

• C: Orthodontically treated individuals without allergies or asthma.

• O: Predisposition of individuals with asthma or allergies to root resorption induced by orthodontic treatment. As a secondary outcome, the type of malocclusion, time of orthodontic treatment, and the evaluated dental elements were considered.

• S: Prospective and retrospective observational cohort and case-control studies.

The exclusion criteria included patients with root fractures; dental anomalies regarding number or form, agenesis, incomplete rhizogenesis, microdontia, and taurodontism; previous orthodontic treatment; and other systemic diseases.

### Information sources

Searches were conducted in the databases: PubMed, Scopus, Web of Science, LILACS, Embase, LIVIVO, Google Scholar, and OpenGrey. The search strategies are shown in Table 1 and were carried out until September 26, 2020, without restrictions regarding the date or language of publication. The reference lists of the included studies were searched manually. An alert was created for new studies compatible with the search strategy in the databases.

**Table 1:** Search strategies in the database

Database	Keywords	Results
Pubmed	((((((((((((“asthma”[MeSH Terms] OR “asthma”[All Fields]) OR “asthmas”[All Fields]) OR “asthma s”[All Fields]) OR (((“asthma”[MeSH Terms] OR “asthma”[All Fields]) OR (“bronchial”[All Fields] AND “asthma”[All Fields])) OR “bronchial asthma”[All Fields])) OR ((“respiratory tract diseases”[MeSH Terms] OR ((“Respiratory”[All Fields] AND “tract”[All Fields]) AND “diseases”[All Fields])) OR “respiratory tract diseases”[All Fields])) OR ((“rhinitis”[MeSH Terms] OR “rhinitis”[All Fields]) OR “rhinitides”[All Fields])) OR ((((“rhinitis, allergic”[MeSH Terms] OR (“rhinitis”[All Fields] AND “allergic”[All Fields])) OR “allergic rhinitis”[All Fields]) OR (“rhinitis”[All Fields] AND “allergic”[All Fields])) OR “rhinitis allergic”[All Fields])) OR ((“respiration disorders”[MeSH Terms] OR (“respiration”[All Fields] AND “disorders”[All Fields])) OR “respiration disorders”[All Fields])) OR (((((((“allergie”[All Fields] OR “hypersensitivity”[MeSH Terms]) OR “hypersensitivity”[All Fields]) OR “allergies”[All Fields]) OR “allergy”[All Fields]) OR “allergy and immunology”[MeSH Terms]) OR (“allergy”[All Fields] AND “immunology”[All Fields])) OR “allergy and immunology”[All Fields])) OR ((((“respiratory tract infections”[MeSH Terms] OR ((“Respiratory”[All Fields] AND “tract”[All Fields]) AND “infections”[All Fields])) OR “respiratory tract infections”[All Fields]) OR (“Respiratory”[All Fields] AND “infection”[All Fields])) OR “respiratory infection”[All Fields])) OR ((((“respiratory tract infections”[MeSH Terms] OR ((“Respiratory”[All Fields] AND “tract”[All Fields]) AND “infections”[All Fields])) OR “respiratory tract infections”[All Fields]) OR (“Respiratory”[All Fields] AND “infections”[All Fields])) OR “respiratory infections”[All Fields])) OR (“Respiratory”[All Fields] AND ((((“change”[All Fields] OR “changed”[All Fields]) OR “changes”[All Fields]) OR “changing”[All Fields]) OR “changings”[All Fields]))) AND ((((((((“plant roots”[MeSH Terms] OR (“plant”[All Fields] AND “roots”[All Fields])) OR “plant roots”[All Fields]) OR “root”[All Fields]) AND “resorp*”[All Fields]) OR ((((“plant roots”[MeSH Terms] OR (“plant”[All Fields] AND “roots”[All Fields])) OR “plant roots”[All Fields]) OR “root”[All Fields]) AND ((((“shorten”[All Fields] OR “shortened”[All Fields]) OR “shortening”[All Fields]) OR “shortenings”[All Fields]) OR “shortens”[All Fields]))) OR ((((“apical”[All Fields] OR “apically”[All Fields]) OR “apicals”[All Fields]) OR “apices”[All Fields]) AND ((((“resorption”[All Fields] OR “resorptional”[All Fields]) OR “resorptions”[All Fields]) OR “resorptive”[All Fields]) OR “resorptives”[All Fields]))) OR “OIRR”[All Fields]) OR ((“tooth resorption”[MeSH Terms] OR (“tooth”[All Fields] AND “resorption”[All Fields])) OR “tooth resorption”[All Fields]))	120
Scopus	((ALL (asthma OR “Respiratory tract disease” OR “Allergic rhinitis” OR rhinitis OR “Rhinitis, Allergic” OR “Respiration Disorders” OR “Respiratory diseases” OR “Respiratory changes” OR “Respiratory infection” OR “Respiratory infections” OR allergy OR allergic OR “Allergy asthma” OR “Bronchial Asthma”)) AND (TITLE-ABS-KEY (“Root resorption” OR “Resorption, root” OR “Tooth resorption” OR “Root Resorp*” OR “Root Shortening” OR “Apical Resorption” OR oirr)))	109
Web of Science	ALL=(asthma OR Respiratory tract disease OR Allergic rhinitis OR rhinitis OR Rhinitis, Allergic OR Respiration Disorders OR Respiratory diseases OR Respiratory changes OR Respiratory infection OR Respiratory infections OR allergy OR allergic OR Allergy asthma OR Bronchial Asthma) AND ALL=(Root resorption OR Resorption, root OR Tooth resorption OR Root Resorp* OR Root Shortening OR Apical Resorption OR oirr)	56
LILACS	(mh:(asthma)) OR (mh:(asma)) OR (mh:(asthma,bronquial)) OR (mh:(respiratory tract diseases)) OR (mh:(asma bronquial)) OR (mh:(respiratory tract disease)) OR (tw:(enfermedades tracto respiratório)) OR (mh:(rhinitis)) OR (tw:(rinitis)) OR (mh:(rhinitis allergic)) OR (mh:(respiration disorders)) OR (tw:(desórdenes respiratórios)) OR (tw:(allergy)) AND (mh:(Root resorption)) OR (mh:(Reabsorción radicular)) OR (tw:(Root shortening)) OR (tw:(Acortamiento de la raiz)) OR (tw:(OIRR)) OR (tw:(Apical resorption)) OR (tw:(Tooth resorption)) OR (tw:(Reabsorción dental))	119
Embase	(“asthma”/exp OR “asthmatic state”/exp OR “respiratory tract disease”/exp OR “rhinitis”/exp OR “allergic rhinitis”/exp OR “breathing disorder”/exp OR “allergy”/exp OR “respiratory infections”/exp) AND (“root resorption” OR “root shortening or” OR “apical resorption or tooth resorption”)	19
LIVIVO	(Asthma OR Bronchial Asthma OR Respiratory Tract Diseases OR Rhinitis OR Rhinitis Allergic OR Respiration Disorders OR Allergy OR Respiratory Infection OR Respiratory Infections OR Respiratory Changes) AND (Root Resorp* OR Root Shortening OR OIRR OR Apical Resorption OR Tooth Resorption)	82
Google Scholar	(Asthma OR Rhinitis OR Allergy AND Root Resorption OR Root Shortening OR OIRR)	200
OpenGrey	Root resorption	9

### Search strategy and study selection

Two independent examiners (C.S and S.B) screened the titles and/or abstracts of studies retrieved from the searches to identify the inclusion criteria. In cases of disagreement, a third examiner was consulted (D.N). The search strategy was developed from a combination of MeSH, entry-terms, and keywords related to the PECOS strategy using Boolean operators. The selected articles were exported to a reference manager (EndNote®, Clarivate Analytics, Philadelphia, USA) for the removal of duplicates and to exclude those that did not meet the pre-established inclusion criteria. Finally, the relevant articles were read for the final selection and a third examiner was consulted (D.N) to resolve discrepancies.

### Data collection process and summary measures

The same reviewers performed data extraction independently. Data were collected based on the following items: authorship, including author names, year of publication and study design; sample characteristics, sample size, distribution by sex, and average age; characteristics of malocclusion, orthodontic appliance, and duration of orthodontic treatment; exposure to the allergy or asthma risk factor; methodology including teeth evaluated and evaluation method; results, including the amount of root resorption and the prevalence of risk factors, in addition to the odds ratio of individuals with allergies or asthma to develop root resorption; and study conclusions.

### Risk of bias in individual studies

The analysis of the risk of bias (RoB) of the selected studies was carried out through the checklists for critical evaluation from the Joanna Briggs Institute for cohort and case-control studies (https://joannabriggs.org/). The goal of critical appraisal (assessment of the risk of bias) is to assess the methodological quality of a study and to determine the extent to which a study has excluded or minimized the possibility of bias in its design, conduct, and analysis. The critical analysis corresponds to the completion of checklists with 10 questions with answers “Yes,” “No,” “Not clear,” and “Not applicable.” The evaluators agreed on the scoring criteria prior to conducting the critical analysis. Thus, the studies were characterized as high RoB when up to 49% of the answers were “YES,” moderate risk when between 50 and 69% of the answers were “YES,” and low when more than 70% of the answers were “YES,” regardless of the question asked. Two examiners independently evaluated the RoB of the selected studies (C.S and S.B) and in the case of discrepancies, a third examiner was consulted (D.N).

### Level of evidence

The outcomes evaluated using the GRADE tool were classified based on the predisposition of patients with asthma or allergies to OIIRR. The studies were evaluated based on the study design, RoB, inconsistency, indirect evidence, and imprecision.

## Results

### Study selection

**Fig. 1:**
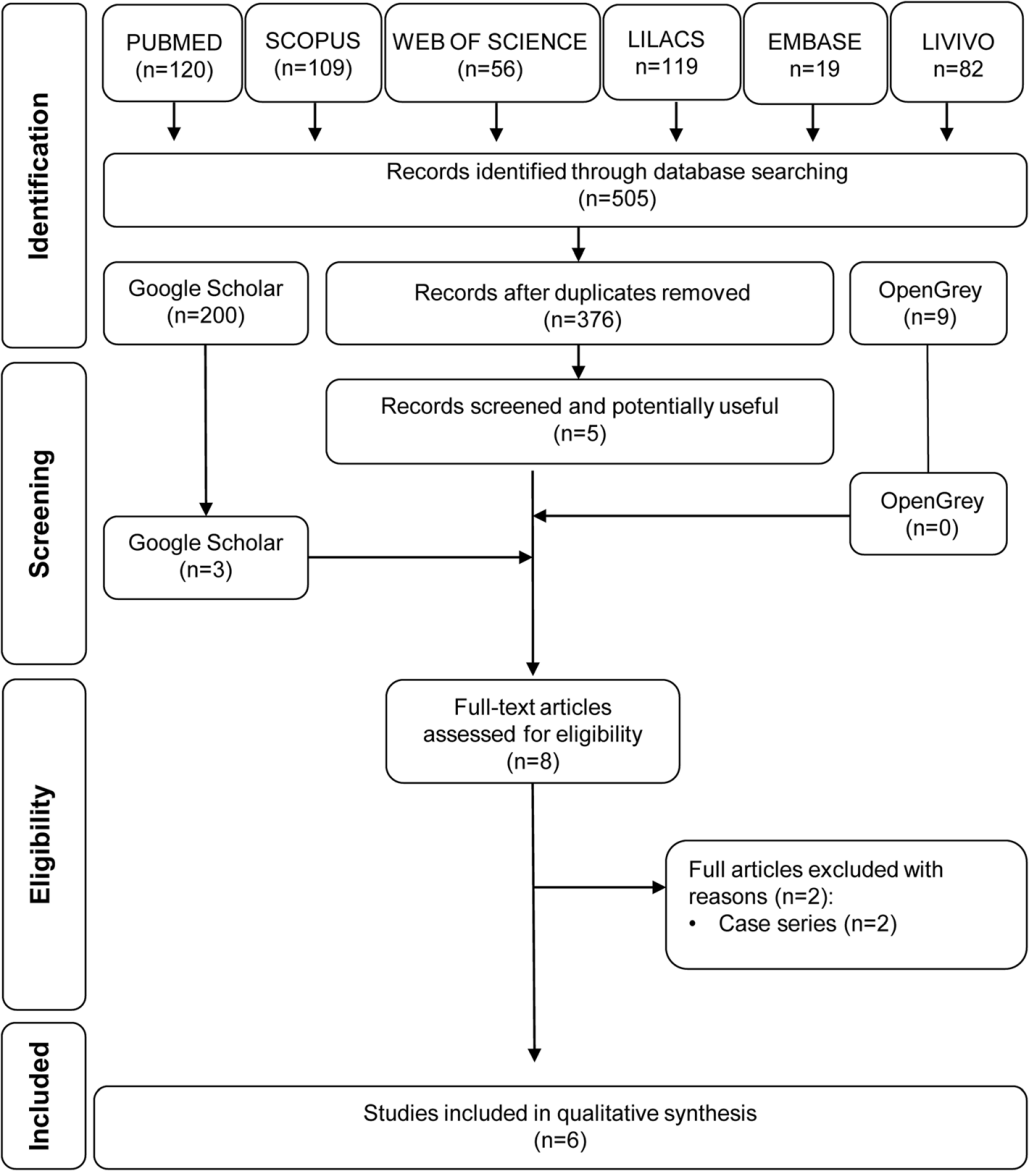
PRISMA flow diagram of article retrieval

The database searches found 505 references: PubMed (*n* = 120), Scopus (*n* = 109), Web of Science (*n* = 56), Lilacs (*n* = 119), Embase (*n* = 19), and Livivo (*n* = 82). After the removal of duplicate references using EndNote® manager, 376 articles remained. After reading titles and abstracts, five potentially selectable studies remained. The search in the gray literature found 209 references: Google scholar (*n* = 200) and OpenGrey (*n* = 9). From the gray literature, three studies were selected after reading titles and abstracts. Thus, eight studies were selected for reading the texts in full and applying the eligibility criteria, which resulted in the exclusion of two case series studies [4, 18]. Six studies were selected for qualitative analysis [11, 14–17, 19]. The process of identification, selection, and exclusion of studies is shown in the PRISMA flow diagram of article retrieval (Fig. 1).

### Study characteristics

The six included studies were observational and retrospective of which one was a cohort type [14] and aimed to determine whether individuals with asthma had a higher incidence of root resorption. Five were case-control studies [11, 15–17, 19], among which, one study observed the influence of asthma on the degree of root resorption [15], two evaluated the association between the allergy risk factor and root resorption [17, 19]; one assessed the association between root resorption and risk factors for allergies and asthma [11]; and the other verified the prevalence of immune diseases in individuals who underwent orthodontic treatment and expressed root resorption [16].

Thus, four studies evaluated patients with allergies [11, 16, 17, 19] and four studies included individuals with asthma in their samples [11, 14–17, 19]. The mean average age ranged between 13.9 (± 1.8) [14] and 17.7 (± 5.1) years [11]. The average time of orthodontic treatment ranged from 1.8 (± 0.4) to 3.1 (± 1.19) years [11, 14]. The cohort study [14] showed a sample of 141 individuals. The sample sizes of the case-control studies varied between 50 [17] and 683 [15] individuals. Two studies did not report the classification of their samples based on malocclusion [14, 17]. All studies were performed on individuals with fixed appliances in both arches. Concerning the teeth evaluated for the level of root resorption, two studies included maxillary premolars [13, 19], two—the maxillary and mandibular incisors [15, 19], one—the mesial- and distal roots of the maxillary 1st molars [14], and two studies evaluated all teeth [11, 16].

There was great methodological heterogeneity among the included studies regarding the methods of evaluation and diagnosis of root resorption. The evaluation was carried out through panoramic radiography in three studies [11, 14, 16], periapical radiographs in two [15, 19], and histological sections in one study [17]. Two studies used the Levander and Malmgren [20] method to measure root resorption [15, 19], one [14] used the Sharpe Scale [21] method, one used a digital caliper to measure the distance from the cementum-enamel junction to the root apex [11], one carried out the histological analysis of the resorption areas measuring the length and depth of the resorbed area [17], and one study measured the root length using panoramic radiographs and determined that individuals with up to 25% of resorption did not have root resorption [16]. The summary of data from the included studies is available in Table 2.

**Table 2:** Summary of the data from included studies

1. Authorship	2. Material				3. Exposure	4. Methodology	5. Results			6. Conclusions
Author, year Study design	Risk factor/outcome M/F (***n***) Mean age ± SD (years)		Malocclusion (Mocl) Orthodontic device Mean treatment time of orthodontic treatment ± SD (years)		Asthma or allergies	Evaluated teeth Evaluation method	Incidence of root resorption (%)/prevalence of risk factors (%)		Odds ratio (OR) (IC 95%) ***p*** value	
	Control	Exposed	Control	Exposed			Control	Exposed		
McNab et al. [14], 1999 Retrospective cohort	Patients without asthma 38M/59F 13.9 ± 1.8 years	Patients with asthma 18M/26F 14.5 ± 3.2 years	Mocl: N.I Fixed appliance, with or without headgear 1.9 ± 0.5 years	Mocl: NI Fixed appliance, with or without headgear 1.8 ± 0.4 years	Asthma	PM’s, mesio-buccal and disto-buccal roots of the upper 1st M, mesial and distal roots of the lower 1st M Panoramic radiography Sharpe scale	T0: Severe OIIRR: 0.93% T1: Severe OIIRR: 15.27% N. I	T0: Severe OIIRR: 2.3% T.1: Severe OIIRR: 13.15% N. I	OR=N.A Multivariate analysis: *p*=0.019	- Although there is an association between root resorption and the asthma risk factor (*p* = 0.019), asthmatic and non-asthmatic individuals exhibited similar values of severe OIIRR.
Melo et al. [15], 2018 Case-control	Patients with initial OIIRR 300M/314F 14.37 ± 2.76 years	Patients with severe OIIRR 32M/37F 15.09 ± 3.44 years	Mocl: Cl I=266 Cl II=319 Cl III=29 Fixed appliance 2.07 ± 0.93 years	Mocl: Cl I=24 Cl II=43 Cl III=2 Fixed appliance 2.72 ± 1.07 years	Asthma	Upper and lower incisors Periapical radiography Levander and Malmgren method	N.I With asthma=35% Without asthma= 65%	N.I With asthma=36.2% Without asthma=63.7%	OR= 1.05 (0.62-1.77) *p*=0.94	-The prevalence of individuals with asthma was like the two groups. -There was no association between the risk factor asthma and OIIRR (*p* = 0.841) -Asthmatic and non-asthmatic individuals have the same chance of developing OIIRR (OR=1.05, 95% CI=0.62-1.77).
Nishioka et al. [11], 2006 Case-control	Patients without OIIRR 18M/42F M: 15.9 **±** 4.5 years F: 18.5 **±** 5.2 years 17.7 ± 5.1 years	Patients with OIIRR 18M/42F M: 17.7 **±** 5.7 years F: 16.4 **±** 6.0 years 16.8 ± 5.9 years	Mocl: Cl I=10 Cl II=29 Cl III=21 Fixed appliance 2.96 ± 0.56 years	Mocl: Cl I=10 Cl II=29 Cl III=21 Fixed appliance 3.10 ± 1.19 years	Allergies and asthma	All teeth Panoramic radiography, measured with a digital pachymeter	N.I With allergies: 21% With asthma: 5%	N.I With allergies: 40% With asthma: 15%	**Allergy risk factor:** OR= 2.41 (1.08-5.37) *p*=0.04 **Asthma risk factor:** OR=3.35 (0.86-13.06) *p=*0.12	-The prevalence of risk factors was higher in individuals with OIIRR. - Allergic individuals are more likely to develop OIIRR than non-allergic individuals (OR = 2.41, 95% CI = 1.08-5.37). -However, asthmatic, and non-asthmatic individuals have the same chance of developing OIIRR (OR=3.35, 95% CI=0.86-13.06).
Owman-Moll et al. [17], 2000 Case-control	Patients with initial OIIRR 25 (N.I.) 13.4 a ±N. I	Patients with severe OIIRR 25 (N.I.) 13.4 a ±N. I	Mocl: N. I. Fixed appliance and lower lingual arch N. I	Mocl: N. I. Fixed appliance and lower lingual arch N. I	Allergy	Upper PM’s Histological analysis	N.I With allergies: 13.3%	N.I With allergies: 26.6%	OR=1.17 (0.38-3.75) *p*=1	- The prevalence of allergies was higher in individuals with severe OIIRR. -However, allergic, and non-allergic individuals have the same chance of developing OIIRR (OR=1.17, 95% CI=0.38-3.75).
Pastro et al. [19], 2018 Case-control	Patients with initial OIIRR 252M/255F 14.21 ± 2.45 years	Patients with severe OIIRR 40M/53F 14.57 ± 2.67 years	Mocl: Cl I=220 Cl II=269 Cl III=18 Fixed appliance 1.81 ± 0.83 years	Mocl: Cl I=38 Cl II=52 Cl III=3 Fixed appliance 2.41 ± 0.99 years	Allergy	Upper and lower incisors Periapical radiography Levander and Malmgren method	N.I With allergies: 42% Without allergy:58%	N.I With allergies: 49.46% Without allergy: 50.53%	OR=1.35 (0.86-2.1) *p*=0.22	-The prevalence of allergies was higher in individuals with severe OIIRR. -There was no association between the allergy risk factor and OIIRR (*p* = 0.182). -Allergic and non-allergic individuals have the same chance of developing OIIRR (OR=1.35, 95% CI=0.86-2.1).
Shim et al. [16], 2003 Case-control	Patients without OIIRR 25M/26F 15.40 ± 4.10 years	Patients with OIIRR 25M/26F 16.10 ± 3.30 years	Mocl: Cl I=15 Cl II=13 Cl III=23 Fixed appliance 2.31 ± 0.77 years	Mocl: Cl I=15 Cl II=14 Cl III=22 Fixed appliance 2.13 ± 0.73 years	Allergy and asthma	All teeth Panoramic radiography Measurement of root length on panoramic radiography	N.I With allergies: 31% With asthma: 5.88%	N.I With allergies: 49% With asthma: 17.64%	**Allergy risk factor:** OR= 2.10 (0.93-4.71) *p*=0.1 **Asthma risk factor:** OR=3.42 (0.87-13.5) *p=*0.12	-The prevalence of allergies was higher in individuals with OIIRR, but without statistical significance (OR=2.1, 95%CI= 0.93-4.71). -The prevalence of asthma was statistically higher in individuals with OIIRR (*p* = 0.01). -However, asthmatic individuals have the same chance of developing OIIRR as non-asthmatic individuals (OR = 3.42, 95%CI = 0.87-13.5).

*T0* before orthodontic treatment; *T1* after orthodontic treatment; *M* molar; *PM* premolar, *NI* not informed; *N.A* not applied; *SD* standard deviation; *M* male; *F* female; *OIIRR* orthodontically induced inflammatory root resorption

### Results of individuals studies

Of the four studies [11, 16, 17, 19] that evaluated patients with allergies, three [16, 17, 19] reported that although the prevalence of allergies is higher among individuals with root resorption, individuals with allergies have the same chance of developing OIIRR as individuals with no allergies. Only one study considered allergies as a risk factor for the development of root resorption after orthodontic treatment [11]. Likewise, the four studies [11, 14–16] in asthmatic individuals state that they have the same chance of OIIRR as non-asthmatics, although the prevalence of asthma was higher in groups of individuals with considerable root resorption. The cohort study [14] reported that while there is an association between root resorption and allergies (*p* = 0.019), the level of severe resorption was similar between individuals with and without asthma.

### Synthesis of result

A meta-analysis was not performed due to the considerable methodological differences between the studies regarding the teeth evaluated, the sample units, and the methods of diagnosis and measurement of root resorption. To complement the findings of the case-control studies, the odds ratio of the individuals with allergies/asthma was calculated. The results can be seen in Table 2.

Only one study [11] reported a greater chance of individuals with allergies developing OIIRR, OR = 2.41, *p* = 0.04. Three studies [11, 15, 16] demonstrated that individuals with asthma have a similar chance compared to non-asthmatic individuals of developing OIIRR, OR = 1.05 to 3.42, *p* = 0.12 to 0.94. The odds ratio for one study was not calculated because it is a cohort study [14].

### Risk of bias within studies

Of the four studies that evaluated patients with allergies [11, 16, 17, 19], two were classified with low RoB [11, 19], one with high [17], and one with a moderate RoB [16]. Three studies evaluating individuals with asthma presented low RoB [11, 14, 15], and one moderate [16]. The RoB was related to unreported [8, 15] and uncontrolled [11, 15, 17] confounding factors, to the use of imprecise methods of measuring root resorption [11, 14, 16], and to the absence of an appropriate statistical analysis including regression models to adjust for confounding factors [16, 17]. Tables 3 and 4 show the evaluation of the RoB of the included studies.

**Table 3:** RoB of case control studies in the qualitative synthesis based on the Joanna Briggs Institute Critical Appraisal Checklist

Questions—analytical case control studies	Melo et al. [15]	Nishioka et al. [11]	Owman-Moll et al. [17]	Pastro et al. [19]	Shim et al. [16]
1—Were the groups comparable other than the presence of disease in cases or the absence of disease in controls?	Y	Y	U	Y	Y
2—Were cases and control matched appropriately?	Y	Y	U	Y	Y
3—Were the same criteria used for identification of cases and controls?	Y	Y	Y	Y	Y
4—Was exposure measured in a standard, valid, and reliable way?	Y	Y	Y	Y	Y
5—Was exposure measured in the same way for cases and controls?	Y	U	Y	Y	Y
6—Were confounding factors identified?	U	Y	U	U	U
7—Were strategies to deal with confounding factors stated?	N	N	N	N	U
8—Were outcomes assessed in a standard, valid, and reliable way for cases and controls?	Y	N	Y	Y	N
9—Was the exposure period of interest long enough to be meaningful?	Y	Y	U	Y	Y
10—Was appropriate statistical analysis used?	Y	Y	U	Y	U
%Yes/risk	80.0	70.0	40.0	80.0	60.0
Overall	Low	Low	High	Low	Moderate

*Y* yes, *N* no, *U* unclear, *N.A* not applicable

**Table 4:** RoB of cohort study in the qualitative synthesis based on the Joanna Briggs Institute Critical Appraisal Checklist

Questions—analytical cohort study	McNab et al. [14]
1—Were the two groups similar and recruited from the same population?	Y
2—Were the exposures measured similarly to assign people to both exposed and unexposed groups?	Y
3—Was the exposure measured in a valid and reliable way?	Y
4—Were confounding factors identified?	Y
5—Were strategies to deal with confounding factors stated?	Y
6—Were groups/participant free of the outcome at the start of the study (or at the moment of exposure)?	Y
7—Were the outcomes measured in a valid and a reliable way?	N
8—Was the follow-up time reported and sufficient to be long enough for outcomes to occur?	U
9—Was follow-up complete, and if not, were the reasons for loss to follow up described and explored?	Y
10—Were strategies to address incomplete follow-up utilized?	U
11—Was appropriate statistical analysis used?	Y
%Yes/risk	72.7
Overall	Low

*Y* yes, *N* no, *U* unclear, *N.A* not applicable

### Assessment of the certainty of evidence

The certainty of evidence that individuals with allergies have the same predisposition to OIIRR as individuals with no allergies was low. Among the four included studies, one had a high RoB [17], one moderate [16], and two low RoB [11, 19]. In addition to the fact that they are observational studies, which reduces the level of certainty of the evidence, they have limitations in the identification [16, 17, 19] and control of confounding factors [11, 16, 17] and in the accuracy of the method of evaluation of root resorption [11, 16]. Similarly, the evidence that asthmatic individuals have the same predisposition to OIIRR as non-asthmatic was judged as low. Among the four studies evaluated, one had moderate RoB [16] and the other low RoB [11, 14, 15]. The low level of certainty of evidence was justified by the methodological differences between the studies related to the study designs, where one study was a cohort [14] and the others were case-control studies [11, 15, 16], associated with the lack of control of confounding variables [11, 15, 16] and the use of panoramic radiographs for the diagnosis of root resorption in three studies [11, 14, 16]. The assessment of the certainty of evidence according to GRADE is described in Table 5.

**Table 5:** Evaluation of the level of certainty of the evidence by the GRADE PRO tool

Certainty assessment							Impact	Certainty	Importance
№ of studies	Study design	Risk of bias	Inconsistency	Indirectness	Imprecision	Other considerations			
**Predisposition of allergic patients to orthodontically induced root resorption**									
4	Observational studies	Serious ^a^	Not serious	Not serious	Not serious	All plausible residual confounding would suggest spurious effect, while no effect was observed.	Of the 4 studies included, one has a high RoB [17] and one a moderate risk [16]. Studies have limitations in the identification and control of confounding factors. Three studies state that allergy is not a risk factor for root resorption [16, 17], while a study with a low RoB states that allergy is a risk factor for orthodontically induced root resorption [11]	⨁⨁◯◯ LOW	IMPORTANT
**Predisposition of asthmatic patients to orthodontically induced root resorption**									
3	Observational studies	Serious ^b^	Serious ^c^	Not serious	Not serious	Strong association. All plausible residual confounding would suggest spurious effect, while no effect was observed	Among the 3 studies evaluated, one has a moderate RoB [16] and the others have a low RoB [11, 15]. In addition to the lack of control of confounding variables, there is inconsistency between the results demonstrated by the value of the methodological heterogeneity between the studies. All studies conclude that asthma is not a risk factor for root resorption.	⨁⨁◯◯ LOW	IMPORTANT

^a^One study has high RoB [17], one has moderate [16], while the others have low [11, 19]. All studies are deficient in the identification and control of confounding factors

^b^Two studies have low RoB [11, 15], while one study has moderate RoB [16]. All are deficient in the identification and control of confounding factors

^c^There is considerable methodological heterogeneity between the studies, concerning the teeth evaluated for root resorption, the sample unit, and the methods of diagnosis of resorption

## Discussion

### Summary of evidence

Although it was shown that individuals with allergies or asthma have the same predisposition to OIIRR as individuals without allergies or asthma, the level of certainty of the evidence was low. However, it is important for clinical applicability since these patients are part of the orthodontist’s clinical routine.

Among two studies with low RoB [11, 19], one with moderate [16] and one with high RoB [17], the prevalence of the allergy was higher in individuals with a higher level of root resorption, varying from 26.6% [17] to 49.46% [19]. However, only one of these studies [11] with an estimated allergy prevalence of 40% in the group of individuals with resorption stated that these patients have a greater chance of developing root resorption, where OR = 2.41, 95% CI 1.08-5.37. This fact may be associated with cellular changes in the immune system of allergic or asthmatic individuals since the chemical mediators produced by allergies or asthma can stimulate the cells that trigger the process of root resorption [14].

Regarding the ethnicity of the evaluated population, the only study in this review [11] that associated the presence of allergies with a higher level of root resorption was carried out in a Japanese sample. The literature points to a study carried out in Thailand that reported allergies as a factor associated with OIIRR (*p* = 0.003), but it was not included in the assessment because it was a case series [5]. Contrarily, a previous study [22] showed that Asians have less root resorption than Hispanic or white individuals. Considering that ethnic differences can affect the shape and size of teeth [22], it is plausible that initial root resorption, without clinical significance in teeth with normal dimensions, can result in major damage to teeth with reduced size. The predisposition of Asian individuals to OIIRR can be elucidated with new studies with samples that contain a balanced number of individuals from different ethnicities controlled as a confounding factor.

The diagnosis of allergies based on clinical records can be inaccurate. Also, the studies did not report which types of allergy or allergenic factors were evaluated, which is considered a confounding factor when interpreting the results. There is no homogeneity related to the criteria for the diagnosis of immune diseases between the evaluated studies, which increases the RoB associated with the interpretation of the results. However, even in a study [17] with moderate RoB where the allergic condition was verified with medications taken or medical consultation, no association was observed between allergies and OIIRR.

Three studies [11, 14, 15] with low RoB and one with moderate RoB [16] evaluated the predisposition of individuals with asthma to OIIRR. The three case-control studies [11, 15, 16] demonstrated that there is no greater chance of developing OIIRR associated with the asthma risk factor. The cohort study [14] corroborates this finding because although there is an association between root resorption and asthma (*p* = 0.019), the level of resorption was similar between asthmatic and non-asthmatic individuals. In all studies evaluating individuals with asthma [11, 14, 16], the prevalence of asthma was higher in groups of patients with a higher level of root resorption, ranging from 15% [11] to 36.2% [15]. However, the odds ratio values for each study did not show significant differences between the individuals with asthma compared to the individuals without asthma to develop root resorption (Table 2).

Regarding the duration of orthodontic treatment, in two case-control studies with low RoB [15, 19], where the sample was initially classified based on the level of root resorption in patients with mild and severe resorption, the treatment time was approximately 6 months longer in the group with severe resorption. These results corroborate with the literature and indicate that there is a positive association between OIIRR and treatment time [5].

Class II malocclusion had the highest prevalence in three [11, 15, 19] of the six [11, 14, 17] studies evaluated, ranging from 48.3% [11] to 53.5% [19]. The literature does not indicate an association between the type of malocclusion and the severity of root resorption [4, 19]. However, individuals with class I or II malocclusion and vertical growth have decreased pharyngeal air space compared to individuals who have a normal growth pattern [23], confirming the fact that the width of the air space can be influenced by the craniofacial growth pattern [24]. It is important to emphasize the prevalence of mouth breathing in individuals with nasopharyngeal airway obstruction and class II malocclusion [25]. This is because individuals with asthma or allergies may develop mouth breathing because of breathing difficulties caused by increased airflow resistance due to inflammatory response characteristic of these systemic changes [26]. Still, asthmatic individuals may have a higher prevalence of malocclusion, especially related to a crossbite, overbite, overjet, and crowding [27]; in addition to the decrease in air space, which can change the mandibular position and lip sealing, causing esthetic, functional changes, and malocclusion [28]. Children with allergic rhinitis have increased anterior facial height, increased overjet, deep palate, and decreased intermolar width in the upper arch [29]. Orthodontic treatment in individuals with vertical growth pattern potentially associated with mouth breathing [26], atresic jaws, crossbites, increased overbite or overjet, may require a longer time to perform orthodontic mechanics, and consequently lead to a greater incidence of root resorption. These variables, which can confuse the interpretation of the results, were not identified, or controlled by the evaluated studies. Thus, it is recommended that further studies be carried out to control the variables related to malocclusion and the craniofacial growth pattern of individuals with asthma or allergies.

Two studies with low RoB and larger sample sizes [15, 19] evaluated maxillary and mandibular incisors in more than 500 individuals diagnosed with mild root resorption using periapical radiographs. The results corroborate that incisors are the elements most affected by root resorption [30]. The assessment of the root region is more accurate when performed by periapical radiographs compared to panoramic radiographs, which can overestimate resorption by approximately 20% [3]. The evaluation of root resorption using panoramic radiographs was considered a methodological limitation present in three studies [11, 14, 16]. The methods of evaluation by Levander and Malmgren [20] and Sharpe [21] are methods of qualitative and subjective analysis and there is no one method superior to the other. A study reported that individuals with allergies have a greater chance of developing OIIRR than ones without allergies [11] evaluated all dental elements through panoramic radiographs and measurement in millimeters corresponding to the longitudinal axis of the enamel-cement junction to the root apex. Although this measurement is quantitative, and therefore less subjective than the assessments by predetermined visual scales, panoramic radiographs have limitations in accurately identifying the enamel-cement junction [31]. Finally, the study with high RoB [17] evaluated premolars from longitudinal histological sections where the amount of the extent and depth of the total root resorption area was measured, which seems to be a more accurate assessment than the qualitative measurement methods. Thus, we observe the great methodological differences between the studies regarding the measurement of root resorption, compromising the level of certainty of the evidence generated. This signals the need for further studies with standardized methods of measurement and diagnosis of both root resorption and systemic conditions.

Using the GRADE tool, the certainty of the evidence generated states that patients with asthma or allergies do not have a greater predisposition to OIIRR was classified as low. However, even though it has a low level of certainty, the evidence is considered important for clinical applicability because it addresses immunological conditions that can be manifested by the general population, and consequently, by individuals undergoing orthodontic treatment. Also, the patient should be informed that root resorption can occur because of orthodontic mechanics regardless of the systemic conditions inherent to the individuals. Considering that the magnitude of these orthodontically induced resorptions has an average value of 1 mm [2], they are not contraindications to orthodontic treatment. They can be identified from periodic radiographic examinations, and if necessary, attenuated from interruptions in the activation of orthodontic force [5].

### Limitations

Quantitative synthesis was not possible because of the great methodological heterogeneity among the included studies and resulted in a low level of certainty. New prospective studies with adequate methodological design from a multiple regression model controlling confounding factors may increase the certainty of the evidence regarding the predisposition of patients with asthma or allergies to OIIRR. Also, it is important to establish a previous protocol for the diagnosis of immune changes, which will reduce the RoB associated with the interpretation of the results. Measurements made with panoramic radiographs may have overestimated the amount of root resorption found. Thereby, it is recommended that new assessments should be made with periapical radiographs or, if possible, with cone-beam computed tomography.

## Conclusions

• Scientific evidence with a low level of certainty states that individuals with asthma or allergies do not have a different predisposition to orthodontically induced root resorption when compared to individuals with no asthma or allergies.

• There was no association between the type of malocclusion and the degree of root resorption. However, uniradicular teeth and patients undergoing longer treatment times are more prone to root resorption.

## Data Availability

The authors declare that all data generated or analyzed during this study are included in this published article and its supplementary information files.

## Abbreviations

OIIRR: Orthodontically induced inflammatory root resorption
RoB: Risk of bias
GRADE: Grading of Recommendations Assessment, Development and Evaluation
OR: Odds ratio
T0: Before orthodontic treatment
T1: After orthodontic treatment
M: Molar
PM: Premolar
NI: Not informed
N.A: Not applied
SD: Standard deviation
M: Male
F: Female
